# Complete genome sequence of a putative novel victorivirus from *Ustilaginoidea virens*

**DOI:** 10.1007/s00705-013-1615-9

**Published:** 2013-02-06

**Authors:** Tingting Zhang, Yinhui Jiang, Junbin Huang, Wubei Dong

**Affiliations:** Department of Plant Pathology, College of Plant Science and Technology and the Key Lab of Crop Disease Monitoring and Safety Control in Hubei Province, Huazhong Agricultural University, Wuhan, 430070 Hubei China

## Abstract

*Ustilaginoidea virens* is the causal agent of a serious disease of rice. Here, we report the presence of five dsRNA bands ranging from about 1.2, 1.5, 1.7, and 1.8 to 5.6 kb in an isolate of this fungus from China and the complete sequence of the largest dsRNA segment, putatively representing the genome of a novel virus, designated as *Ustilaginoidea virens* RNA virus 1 (UvRV1), UvRV1, which has a genome length of 5567 bp and has two consecutive open reading frames (ORFs) with a five-nucleotide overlap. Phylogenetic analysis showed that UvRV1 belongs to the genus of *Victorivirus* in the family *Totiviridae*.

**Electronic supplementary material:**

The online version of this article (doi:10.1007/s00705-013-1615-9) contains supplementary material, which is available to authorized users.

## Introduction

Mycoviruses with double-stranded RNA (dsRNA) genomes have been described in a wide range of fungi covering all four phyla of the true fungi: *Chytridiomycota*, *Zygomycota*, *Ascomycota*, and *Basidiomycota*. Mycoviruses are classified into 11 families, and there are many that have not yet been assigned to a family [1]. Members of five of these 11 families, *Totiviridae*, *Partitiviridae*, *Chrysoviridae*, *Reoviridae*, and *Megabirnaviridae*, have double-stranded (ds) RNA genomes encapsidated in rigid virus particles. The type of dsRNA segments observed in fungal isolates can be quite diverse, even in the same fungal species [2]. This diversity can be seen in the number and size of the dsRNA segments detected in a fungal strain, and it could indicate multipartite viral genomes, mixed infections, or even defective products of virus replication [3].

Rice false smut is a worldwide fungal disease, caused by *U. virens* (teleomorph: *Villosiclava virens*) [4–6]. The disease was first reported from the Tirunelveli district of the Tamil Nadu state of India [6]. In recent years, rice false smut has become more epidemic in areas of Asia, America, and Europe, where new hybrid varieties were widely planted, highly efficient cultivation methods were adopted, and chemical fertilizers were extensively used [7–11]. A characteristic trait of rice false smut is the formation of ball-like colonies in spikelets, which begin to appear 10 to 15 days after rice anthesis. The disease significantly reduces grain quality and yield of rice [8]. Yield losses caused by rice false smut disease have been estimated to vary between 0.2 % and 49 % depending on the infection severity and rice variety [11, 12].

At present, there is no record of a double-stranded RNA virus infecting *U. virens*. In this study, we report the presence of dsRNAs in a sample of *U. virens* isolated from rice in China.

## Provenance of the virus material

**Fig. 1:**
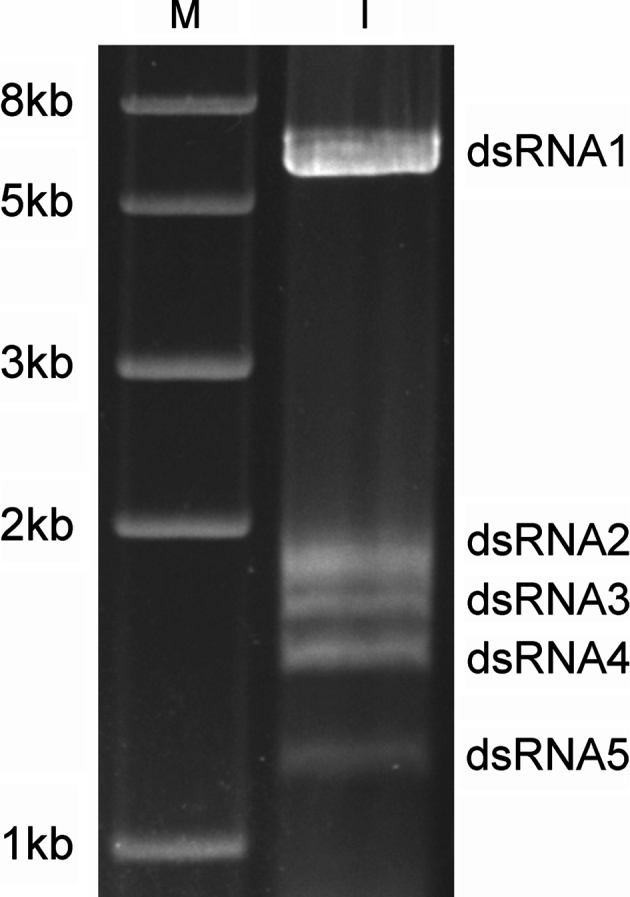
Agarose gel electrophoresis of dsRNA extracted from mycelia of *Ustilaginoidea virens* strain JYH-ZT. The dsRNA preparations were treated with RNase-free DNaseI and S1 nuclease, fractionated on 1.0 % agarose gels and stained with ethidium bromide. Lane M, Trans2 k PlusII DNA Marker (TranGen Biotech, Beijing), used as a size marker; sizes in kb indicated at the *left*. Lane I, five dsRNA elements from *U. virens* are displayed

*Ustilaginoidea virens* strain JYH-ZT, used in this study, was originally isolated from rice cultivated in Hubei, China. This strain was maintained on potato sucrose agar (PSA) plates. For extraction and purification of dsRNAs, mycelia were grown on potato sucrose broth (PSB) with shaking (150 rpm) at 28 °C. dsRNA was extracted from fungal mycelia using CF-11 cellulose (Sigma, St. Louis, MO, USA) column chromatography as described previously [13, 14]. To remove contaminating DNA and ssRNA, we treated the dsRNA sample with RNase-free DNaseI (TAKALA, Dalian, China; RNase-free DNaseI) and S1 nuclease (TAKALA Dalian, China; S1 nuclease) at 37 °C for 30 min. The dsRNA sample was analyzed by 1 % (w/v) agarose gel electrophoresis containing TAE buffer (40 mM Tris-acetate, 2 mM EDTA, pH 8.1) and 500 ng/ml ethidium bromide, and the largest dsRNA fragment, called dsRNA1 (Fig. 1), was extracted from the gel, denatured and used for reverse transcription and PCR amplification. A random-primer amplification method [15] was used to obtain the full-length sequence of dsRNA1. The ends of the molecule were cloned using an improved method [16]. Sequence analysis, alignment, and phylogenetic analysis were performed using DNAMAN, the COBALT web server (http://www.ncbi.nlm.nih.gov/tools/cobalt/cobalt.cgi?link_loc=BlastHomeLink), and the program PhyML 3.0 [17], respectively. The other four dsRNA elements present in the *U. virens* strain JYH-ZT were termed dsRNA2 (1.8 kb), dsRNA3 (1.7 kb), dsRNA4 (1.5 kb), and dsRNA5 (1.2 kb), respectively, according to their sizes (Fig. 1). Their sequences and biological functions are under further investigation.

## Sequence properties

**Fig. 2:**
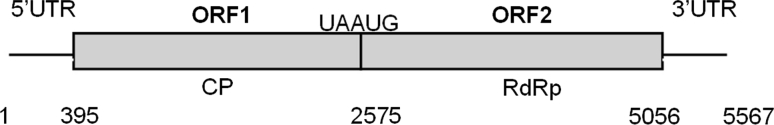
Genome organization of *Ustilaginoidea virens* RNA virus 1 (UvRV1). The 5567-bp genome contained two ORFs. ORF1 encoded a putative CP, and ORF2 encoded a putative RdRp. ORF1 and ORF2 were demarcated by a pentanucleotide, UAAUG, which constituted the stop codon of ORF1 and the start codon of ORF2

The complete sequence of dsRNA1 was 5567 bp and had a GC content of 57 %. Sequence analysis showed that dsRNA1 had two consecutive open reading frames (ORFs) (Fig. 2). ORF1 had a length of 2175 bp and encoded a 725-amino-acid protein (76.189 kDa). ORF2 was 2478 bp in length and encoded an 826-amino-acid protein (91.629 kDa). For the gene sequences, the stop codon of the first gene and the start codon of the following gene overlapped in the coupled termination-reinitiation model of protein expression [18, 19]. Based on this model, we predicted that dsRNA1 contained two ORFs that were demarcated by a pentanucleotide, UAAUG, which constituted the stop codon of ORF1 and the start codon of ORF2. This five-nucleotide overlap has been described in other totiviruses, such as *Aspergillus foetidus* virus slow-1 [20] and *Beauveria bassiana* RNA virus 1 (EMBL/GenBank accession number: CCC42235). The amino acid sequence deduced from ORF1 of dsRNA1 showed a high level of similarity to those of the capsid proteins (CP) of viruses of the family *Totiviridae*, particularly to that of *Beauveria bassiana* RNA virus 1 (BbRV1; 61 % identity). The C-terminus of this putative CP had an Ala/Gly/Pro-rich region, which occurs in mycoviruses of the genus *Victorivirus* [18]. The protein encoded by ORF2 contained conserved motifs characteristic of viral RNA-dependent RNA polymerases (RdRps) and appeared most similar (54 % identity) to an ortholog encoded by BbRV1. The 5’ untranslated region (UTR) in ORF1 had 394 bp and started with a CTTTG sequence, which was the same as the CTTTG motif present in the genome of type II *Trichomonas vaginalis* virus 2 (TVV2) [21], and similar to the CTTAA motif present in the genome of type I *T. vaginalis* virus 1 (TVV1) [22]. The GC content of this region was 52 %. The 3’ UTR in ORF2 had a length of 509 bp and a GC content of 58 %.

**Fig. 3:**
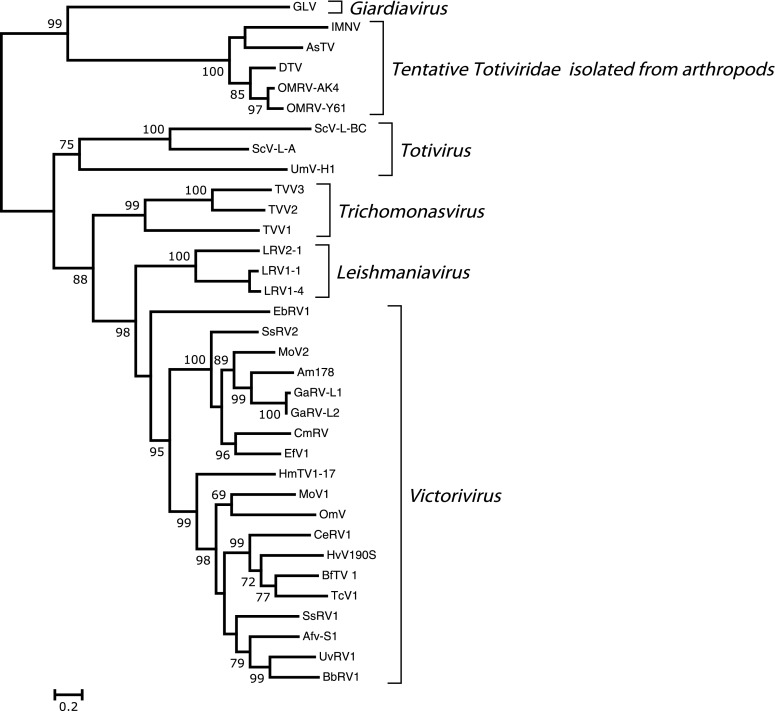
Phylogenetic tree of viruses of the family *Totiviridae* based on RdRp amino acid sequences. The tree was constructed by the maximum-likelihood (ML) method using the program PhyML 3.0 and the LG model of amino acid substitution. The reliability of internal branches was evaluated based on SH-aLRT support. Abbreviations and GenBank accession numbers are as follows: *Aspergillus foetidus* virus slow-1 (AfV-S1; HE588147); *Sphaeropsis sapinea* RNA virus 1 (SsRV1; NP047588); *Beauveria bassiana* RNA virus 1 (BbRV1; CCC42235); *Botryotinia fuckeliana* totivirus 1 (BfTV1; YP001109580); *Tolypocladium cylindrosporum* virus 1 (TcV1; YP004089630); *Helminthosporium victoriae* virus 190S (HvV190S; NP61967); *Magnaporthe oryzae* virus 1 (MoV1; YP122352); *Helicobasidium mompa* totivirus 1-17 (HmTV1-17; NP898833); *Aspergillus* mycovirus 178 (AMV178; ABX7995); *Gremmeniella abietina* RNA virus L2 (GaRV-L2; YP044807); *Gremmeniella abietina* RNA virus L1 (GaRV-L1; AAK11656); *Magnaporthe oryzae* virus 2 (MoV2; YP001649206); *Epichloe festucae* virus 1 (EfV1; CAK02788); *Coniothyrium minitans* RNA virus (CmRV; YP392467); *Sphaeropsis sapinea* RNA virus 2 (SsRV2; NP047560); *Eimeria brunetti* RNA virus 1 (EbRV1; NP108651); *Leishmania* RNA virus 1–1, (LRV1-1; NP041191); *Leishmania RNA* virus 1–4 (LRV1-4; NP619653); *Leishmania* RNA virus 2–1 (LRV2-1; NP043465); *Trichomonas vaginalis* virus 1 (TvV1; AAA62868); *Trichomonas vaginalis* virus 2 (TvV2; AAF29445); *Trichomonas vaginalis* virus (TvV3; NP659390); *Saccharomyces cerevisiae* virus L-A (L1) (ScV-L-A; NP620495); *Saccharomyces cerevisiae* virus L-BC (ScV-L-BC; NP042581); *Ustilago maydis* virus H1 (UmV-H1; U01059); *Giardia lamblia* virus (GLV; NP620070); *Armigeres subalbatus* virus SaX06-AK20 (AsTV; ACH85916); penaeid shrimp infectious myonecrosis virus (IMNV; AAT67231); *Ophiostoma minus* totivirus (OmV; CAJ34336.1); *Chalara elegans* RNA virus 1 (CeRV1; AY561500); *Drosophila melanogaster* totivirus SW-2009a (DTV; GQ342961); Omono River virus, strain AK4 (OMRV-AK4; AB555544); Omono River virus, strain AK4 (OMRV-Y61; AB555545); *Ustilaginoidea virens* RNA virus 1 (UvRV1)

A phylogenetic analysis based on the complete amino acid sequence of putative RdRps encoded by dsRNA1 and totiviruses showed close relationships between dsRNA1 and members of the genus *Victorivirus* (Fig. 3). The phylogenetic analysis showed that this dsRNA element represented a new member of the genus *Victorivirus*, which was called UvRV1. A sequence comparison analysis indicated that the eight conserved motifs of the RdRps sequences of the dsRNA viruses in filamentous ascomycetous fungi [23] were present in this putative RdRp of UvRV1 (Supplementary Fig. 1). Finally, based upon our phylogenetic analysis and the *Victorivirus* species demarcation criteria established by the International Committee on Taxonomy of Viruses, which states that the amino acid sequence identity in pairwise comparisons of CP or RdRp gene products between members of different species is no more than 60 % [24], UvRV1 should be considered a new member of the genus *Victorivirus*. The complete genome sequence has been deposited in the EMBL nucleotide sequence database with the accession number JX524563.

## Electronic supplementary material

Below is the link to the electronic supplementary material.

Supplementary material 1: The eight conserved motifs of the sequences of RdRps of *U. virens* RNA virus 1 (UvRV1). These conserved motifs were constructed using the COBALT web server (http://www.ncbi.nlm.nih.gov/tools/cobalt/cobalt.cgi?link_loc=BlastHomeLink). Abbreviations and GenBank accession numbers are as follows: *Helminthosporium victoriae* virus 190S (HvV190S; NP61967); *Sphaeropsis sapinea* RNA virus 1 (SsRV1; NP047588); *Magnaporthe oryzae* virus 1 (*MoV1*; YP122352); *Saccharomyces cerevisiae* virus L-A (L1) (ScV-L-A; NP620495); *Ustilaginoidea virens* RNA virus 1 (UvRV1). (PDF 44 kb)
