# Supplementary material for: Complete genome sequence of a putative novel victorivirus from Ustilaginoidea virens
Source: Arch Virol. 2013 Feb 6;158(6):1403–6. doi: 10.1007/s00705-013-1615-9 (PMC3668124; doi:10.1007/s00705-013-1615-9)
Supplement: Supplementary file 1 — Supplementary material 1: The eight conserved motifs of the sequences of RdRps of U. virens RNA virus 1 (UvRV1). These conserved motifs were constructed using the COBALT web server (http://www.ncbi.nlm.nih.gov/tools/cobalt/cobalt.cgi?link_loc=BlastHomeLink). Abbreviations and GenBank accession numbers are as follows: Helminthosporium victoriae virus 190S (HvV190S; NP61967); Sphaeropsis sapinea RNA virus 1 (SsRV1; NP047588); Magnaporthe oryzae virus 1 (MoV1; YP122352); Saccharomyces cerevisiae virus L-A (L1) (ScV-L-A; NP620495); Ustilaginoidea virens RNA virus 1 (UvRV1). (PDF 44 kb) [file 705_2013_1615_MOESM1_ESM.pdf]

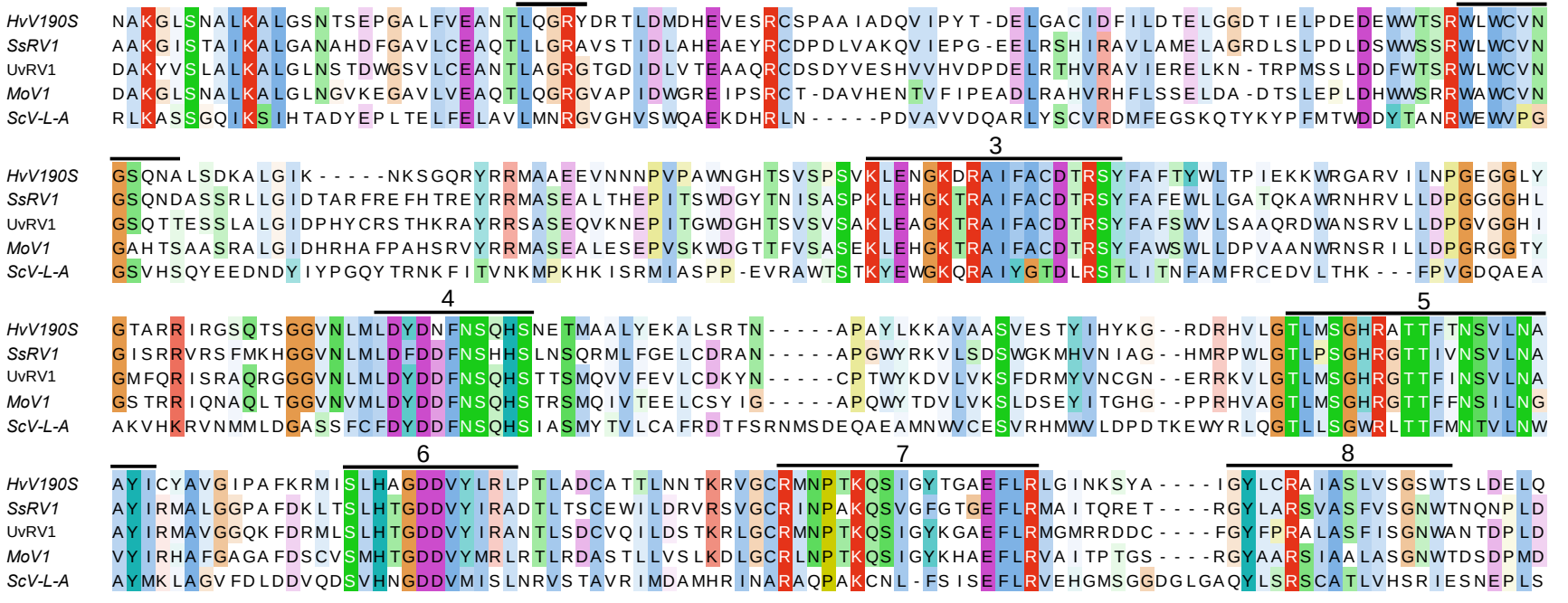
1
2
3
4
5
6
7
8
HvV190S NAKGLSNALKALGSNTSEPGALFVEANTLQGRYDRTLDMDHEVESRCSPAAIADQVIPYT-DELGACIDFILDTELGGDTIELPDEDEWWTSRWLWCVN
SsRV1 AAKGISTAIKALGANAHDFGAVLCEAQTLLGRAVSTIDLAHEAEYRCDPDLVAKQVIEPG-EELRSHIRAVLAMELAGRDLSLPDLDSWWSSRWLWCVN
UvRV1 DAKYVSLALKALGLNSTDWGSVLCEANTLAGRGTGDIDLVTEAAQRCDSDYVESHVVHVDPDELRTHVRAVIERELKN-TRPMSSLDDFWTSRWLWCVN
MoV1 DAKGLSNALKALGLNGVKEGAVLVEAQTLQGRGVAPIDWGREIPSRCT-DAVHENTVFIPEADLRAHVRHFLSSELDA-DTSLEPLDHWWSRRWAWCVN
ScV-L-A RLKASSGQIKSIHTADYEPLTELFELAVLMNRGVGHVSWQAEKDHRLN-----PDVAVVDQARLYSCVRDMFEGSKQTYKYPFMTWDDYTANRWEWVPG
HvV190S GSQNALSDKALGIK-----NKSGQRYRRMAAEEVNNNPVPAWNGHTSVSPSVKLENGKDRAIFACDTRSYFAFTYWLTPIEKKWRGARVILNPGEGGLY
SsRV1 GSQNDASSRLLGIDTARFREFHTREYRRMASEALTHEPITSWDGYTNISASPKLEHGKTRAIFACDTRSYFAFEWLLGATQKAWRNHRVLLDPGGGGHL
UvRV1 GSQTTESSLALGIDPHYCRSTHKRAYRRSASEQVKNEPITGWDGHTSVSVSAKLEAGKTRAIFACDTRSYFAFSWVLSAAQRDWANSRVLLDPGVGGHI
MoV1 GAHTSAASRALGIDHRHAFPAHSRVYRRMASEALESEPVSKWDGTTFVSASEKLEHGKTRAIFACDTRSYFAWSWLLDPVAANWRNSRILLDPGRGGTY
ScV-L-A GSVHSQYEEDNDYIYPGQYTRNKFITVNKMPKHKISRMIASPP-EVRAWTSTKYEWGKQRAIYGTDLRSTLITNFAMFRCEDVLTHK---FPVGDQAEA
HvV190S GTARRIRGSQTSGGVNLMLDYDNFNSQHSNETMAALYEKALSRTN-----APAYLKKAVAASVESTYIHYKG--RDRHVLGTLMSGHRATTFTNSVLNA
SsRV1 GISRRVRSFMKHGGVNLMLDFDDFNSHHSLNSQRMLFGELCDRAN-----APGWYRKVLSDSWGKMHVNIAG--HMRPWLGTLPSGHRGTTIVNSVLNA
UvRV1 GMFQRISRAQRGGGVNLMLDYDDFNSQHSTTSMQVVFEVLCDKYN-----CPTWYKDVLVKSFDRMYVNCGN--ERRKVLGTLMSGHRGTTFINSVLNA
MoV1 GSTRRIQNAQLTGGVNVMLDYDDFNSQHSTRSMQIVTEELCSYIG-----APQWYTDVLVKSLDSEYITGHG--PPRHVAGTLMSGHRGTTFFNSILNG
ScV-L-A AKVHKRVNMMLDGASSFCFDYDDFNSQHSIASMYTVLCAFRDTFSRNMSDEQAEAMNWVCESVRHMWVLDPDTKEWYRLQGTLLSGWRLTTFMNTVLNW
HvV190S AYICYAVGIPAFKRMISLHAGDDVYLRLPTLADCATTLNNTKRVGCRMNPTKQSIGYTGAEFLRLGINKSYA----IGYLCRAIASLVSGSWTSLDELQ
SsRV1 AYIRMALGGPAFDKLTSLHTGDDVYIRADTLTSCEWILDRVRSVGCRINPAKQSVGFGTGEFLRMAITQRET----RGYLARSVASFVSGNWTNQNPLD
UvRV1 AYIRMAVGGQKFDRMLSLHTGDDVYIRANTLSDCVQILDSTKRLGCRMNPTKQSIGYKGAEFLRMGMRRDDC----FGYFPRALASFISGNWANTDPLD
MoV1 VYIRHAFGAGAFDSCVSMHTGDDVYMRLRTLRDASTLLVSLKDLGCRLNPTKQSIGYKHAEFLRVAITPTGS----RGYAARSIAALASGNWTDSDPMD
ScV-L-A AYMKLAGVFDLDDVQDSVHNGDDVMISLNRVSTAVRIMDAMHRINARAQPAKCNL-FSISEFLRVEHGMSGGDGLGAQYLSRSCATLVHSRIESNEPLS
